# Effects of Self-Perceptions of Aging on Social Disconnectedness and Loneliness in Older Adults

**DOI:** 10.1093/geroni/igab046.143

**Published:** 2021-12-17

**Authors:** Rita Xiaochen Hu

**Affiliations:** Social Work, Ann Arbor, Michigan, United States

## Abstract

Research shows that self-perceptions of aging (SPA) predict physical, mental, cognitive, and emotional well-being in older adults. Few studies have examined SPA’s effects on social well-being. Using data from the 2014–2018 Health and Retirement Study, we examined SPA’s effects on older adults’ social connectedness and loneliness (age 65+, N = 3,808). SPA was measured by eight items. Social connectedness was operationalized by network size, social contact, and social participation. The UCLA Loneliness Scale assessed loneliness. Linear regression results show that more positive SPA is correlated with increased social connectedness (b = 0.05 SE = 0.01 p = 0.0003) and decreased loneliness (b = -0.09 SE = 0.02 p < 0.0001) in four years, controlling for sociodemographic and health characteristics. Further, loneliness is a mediator between SPA and social connectedness. Findings suggest that older adults with negative SPA are at risk of both objective and subjective social isolation.

